# Effects of Photobiomodulation in Patients Presenting with Reticular Pseudodrusen: A Retrospective Observational Case Series Study

**DOI:** 10.3390/medicina58111662

**Published:** 2022-11-17

**Authors:** Hoang Mai Le, Carl-Joe Mehanna, Irene De Rosa, Alexandra Miere, Eric Souied

**Affiliations:** 1 Centre Hospitalier Intercommunal de Créteil, 94 000 Créteil, France; 2 Faculty of Medicine, University Paris-Est Créteil (UPEC), 94 000 Créteil, France

**Keywords:** reticular pseudodrusen, photobiomodulation, age-related macular degeneration

## Abstract

*Background and Objectives*: The purpose of this study is to describe the effects of photobiomodulation on drusen regression with patients presenting with reticular pseudodrusen (RPD). *Materials and Methods*: This study is a retrospective observational case series study including patients presenting with RPD who underwent treatment by photobiomodulation. All patients underwent a complete ophthalmic examination and multimodal imaging prior to treatment, including spectral-domain optical coherence tomography (SD-OCT). Eyes were treated two times per week for six consecutive weeks. Best corrected-visual acuity (BVCA) was measured prior and after treatment for all patients. The number of RPD on the SD-OCT scans centered on the macula and stages of RPD was noted at baseline and 6 months after the first treatment session. *Results*: Five eyes of five patients were included in the study. Mean BCVA did not change 6 months after treatment compared to baseline. Mean number of RPD per eye was 112.60 +/− 48.33 RPD at baseline and 111.6 +/− 49.29 in the same area 6 months after treatment. Changes in RPD distribution according to RPD classification were observed before and after treatment with photobiomodulation. Changes in distribution mostly concerned stages 1 and 3 RPD: Total number of stage 1 RPD was 289 and increased to 324 after treatment. Total number of stage 3 RPD was 97 at baseline and decreased to 67 6 months after treatment. Percentage of stage 1 RPD increased from 46% to 56% after treatment. Percentage of stage 3 RPD decreased from 20% to 13% after treatment. *Conclusions*: Changes in RPD distribution were observed before and after treatment with photobiomodulation. The number of stage 3 reticular pseudodrusen decreased while number of stage 1 reticular pseudodrusen increased after treatment.

## 1. Introduction

Age-related macular degeneration (AMD) is one of the leading causes of blindness in subjects aged more than 50 years old [1,2,3], and its prevalence is predicted to increase over time [4]. It is characterized by progressive retinal degeneration and central visual loss, which could lead to severe visual impairment. Late AMD is characterized by the development of geographic atrophy or macular neovascularization [2,5]. Retinal pigment epithelial degeneration that occurs in AMD is due to multiple and diverse factors, such as inflammatory and genetics factors but also mitochondrial dysfunction and oxidative stress [6,7,8].

Drusen is an early feature of AMD and results from dysfunction of the retinal pigment epithelium–photoreceptors complex. They appear as retinal yellow deposits on fundus examination [9,10]. Drusen with a size < 63 um are considered part of the normal aging process, whereas drusen with a size > 63 um are considered as part of early and intermediate AMD with an increased risk of visual dysfunction [11]. Reticular pseudodrusen (RPD), also termed subretinal drusenoid deposits, is a distinct phenotype of drusen [12] located in the subretinal space. They can be found in AMD: in intermediate AMD, geographic atrophy and neovascular AMD, especially in type 3 MNV [13,14].

They also can be found in other retinal diseases such as Sorsby retinal dystrophy or in patients suffering from pseudoxanthoma elasticum [15,16]. 

They can also be isolated and found in eyes without other retinal diseases [17]. Genetics associations have been described in patients presenting with RPD, notably, in association with the ARMS2 gene and the CFH gene, although genetic variants specifically linked to RPD have yet to be determined [18,19].

Over the past decades, several authors have shown some interest in better understanding the physiopathology and the clinical aspects of RPD, using multimodal imaging [20]. They have been shown to be associated with worse visual and anatomical function in AMD, even in early stages of the disease [20]. Several studies found that even without the presence of neovascularization or atrophy, they tend to alter visual acuity as well as contrast sensitivity [21]. Therefore, an early treatment could be of interest. There is no known approved specific treatment to slow down the evolution from early and asymptomatic stages of AMD into late stages of the disease. 

Querques et al. [22] have evaluated the safety and short-term efficacy subthreshold laser treatment in patients presenting with RPD secondary to dry AMD. Although no changes were observed in terms of visual acuity, the distribution among the changed RPD was modified after subthreshold laser treatment.

photobiomodulation consists of the use of selected wavelengths (ranging from visible light to near-infrared 500–1000 nm) produced by a noncoherent light source or a laser. It is an innovative therapy that has shown some beneficial effects on cellular tissues [23]. 

Indeed, near-infrared spectral light and its absorption by photoreceptors lead to a photochemical reaction on the cellular level, leading to increased respiratory chain function and mitochondrial activity. Photoreceptor performance measured by ATP production and ERG is improved [24]. It also uncouples nitric oxide. The freed nitric oxide also activates mechanisms increasing antioxidant production, antiapoptotic pathways and cellular metabolism. photobiomodulation treatment generates more mitochondrial energy via ATP activity and replication [25]. Moreover, it also increases the production of proteins and RNA. Therefore, it has many beneficial effects on both cellular and clinical aspects in numerous disease states [26]. Improving cell survival and increasing antioxidant production could indeed be of interest in patients suffering from AMD. Recently, it has been used as a treatment of photoreceptor degeneration [27]. In preclinical studies using animal models, photobiomodulation decreased cellular and tissue damage resulting from either laser burn, diabetic retinopathy, retinitis pigmentosa or methanol toxicity [28,29]. In recent clinical studies, it was shown to decrease drusen volume and improve visual acuity compared to sham treatment in patients with dry AMD [30,31].

To our knowledge, drusen regression in terms of number and staging after photobiomodulation in patients presenting with RPD has not been investigated. The purpose of this study is to describe the effects of photobiomodulation on drusen regression with patients presenting with RPD.

## 2. Materials and Methods

This study is a retrospective observational study conducted in the Department of Ophthalmology at the Centre Hospitalier Intercommunal de Créteil, in Créteil, France. 

### 2.1. Study Population

Patients diagnosed with RPD who underwent treatment with photobiomodulation in Centre Hospitalier Intercommunal de Créteil between January 2021 and April 2022 were included in this study. Diagnosis of RPD was made on multimodal imaging, including fundus photographs, infrared and autofluorescence imaging and optical coherence tomography (OCT). Best-corrected visual acuity (BCVA) was noted for all patients.

We excluded patients with concomitant macular neovascularization, geographic atrophy, a concomitant retinal or ophthalmic disease, media opacities, including cataracts or posterior capsule opacification, which might interfere with visual acuity or imaging in the study eye(s) and presence of or history of malignancy within the past 5 years.

### 2.2. Treatment and Imaging Modalities

Eyes included in this study had been treated with photobiomodulation using the LumiThera^®^ Valeda™ Light Delivery System (LumiThera, Inc., Poulsbo, WA, USA) two times per week for six consecutive weeks. LumiThera^®^ Valeda™ Light Delivery System delivers 3 distinct wavelengths, in the near-infrared (850 nm), the red (660 nm) and the yellow (590 nm) range. Total duration of one session treatment was of 250 s in total. The treatment consisted of four phases: The first phase lasted for 35 s, with the patient’s eye open (yellow pulse and near-infrared wavelengths). The second phase lasted for 90 s, with the patient’s eye closed (continuous red wavelengths). The third phase was similar to the first one, with a treatment lasting 35 s, with the patient’s eye open (yellow pulse and near-infrared wavelengths). The last phase lasted for 90 s, with the patient’s eye closed (continuous red wavelengths). Patients had oral supplementation with 840 mg of docosahexaenoic acid (DHA) and 270 mg of eicosapentaenoic acid (EPA) daily, which was started one month before the first treatment session and continued during at least 6 months after the first treatment session. The study followed the tenets of Helsinki, and all patients had signed an informed consent before receiving the treatment.

All patients had a complete ophthalmic examination including measure of the BCVA, slit lamp examination, intraocular pressure and fundus examination on the day of the first treatment session before treatment (day-one, D1). They also had a complete retinal imaging on the same day, including color fundus photographs, infrared (IR 30°) and autofluorescence imaging, spectral domain optical coherence tomography (SD-OCT) scans centered in the macula (Heidelberg Engineering, Heidelberg, Germany, swept-source optical coherence tomography (SS-OCT) (Solix, Optovue, CA, USA)). SS-OCTA was performed in order to eliminate any quiescent macular neovascularization. 

The appearance of drusen were analyzed by 2 expert readers (HML and IDR) using SD-OCT images before (D1) and 6 months after treatment: the number of RPD on the dense scan centered on the macula (49 B scans, 5.9 × 5.9 mm, distance between B scans 122 um), and stages of RPD as previously described were noted [22,32]. In cases in which the two readers (HML and IDR) did not agree, the final decision was made by a third expert (CJM). As previously reported [32], stage 1 RPD is defined by diffuse deposition of hyper-reflective material between the retinal pigment epithelium and the inner/outer segments’ boundary. Stage 2 is defined by alteration of the inner/outer segments’ boundary due to accumulation of material. Stage 3 is defined by a thicker and conical shape of accumulated material passing through the inner/outer segments’ boundary. Stage 4 is defined by fading of the accumulated material due to the reabsorption and migration within the inner retinal layers. BCVA was also compared before (D1) and 6 months after treatment.

Morphological changes such as central macular thickness (CMT) and subfoveal choroidal thickness were measured on SD-OCT before (D1) and 6 months after treatment.

### 2.3. Descriptive Analysis

Due to the low number of patients, statistical analysis was not performed. Descriptive analysis was performed using Excel, Microsoft., Version 16.16.27, USA, WA Measures of BCVA expressed in Snellen were converted into Logarithm of the Minimum Angle of Resolution (LogMar) units. Quantitative variables, such as BCVA and number of RPD per eye were expressed by their mean +/− standard deviation and were measured before (D1) and 6 months after treatment. Total number of RPD and percentage of drusen were also measured before and after treatment according to staging.

Ethical approval: the study was approved by the Ethics Committee of the Federation France Macula (2022-41, 17 June 2022).

## 3. Results

Five eyes of five patients were included in the study. There were three males and two females. Mean age of the patients was 82.20 +/− 6.34. Among the included patients, two patients had macular neovascularization in the contralateral eye. At baseline, BCVA was between 20/25 and 20/40 Snellen equivalent for all patients. Mean visual acuity expressed in LogMAR was 0.22 +/− 0.13 (20/32–20/40 Snellen equivalent) at baseline (D1) and 0.20 +/− 0.12 in LogMAR (Snellen equivalent 20/32) 6 months after treatment. Using SD-OCT, we identified a mean of 112.60 +/− 48.33 RPD per eye in the macular area at baseline (D1) located within the macular SD-OCT dense scan (49 B scans, 5.9 × 5.9 mm, distance between B scans 122 um). Six months after treatment, mean number of RPD per eye was 111.6 +/− 49.29 in the same area. Interestingly, the distribution of RPD among the RPD stages changed after the treatment. Total number of stage 1 RPD was 289 and increased to 324 after treatment. Mean number of stage 1 RPD per eye increased from 57.8 +/− 45.77 to 64.8 +/− 44.86 after treatment. 

Total number of stage 2 RPD was 149 at baseline (D1) and 137 6 months after treatment. Mean number of stage 2 RPD per eye was 9.80 +/− 29.21 at baseline and 27.40 +/− 30.81 6 months after treatment.

Total number of stage 3 RPD was 97 at baseline (D1) and decreased to 67 6 months after treatment. Mean number of stage 3 RPD per eye was 19.4 +/− 8.11 before treatment and decreased to 13.40 +/− 10.09 6 months after treatment.

Total number of stage 4 RPD was 28 before and 30 6 months after treatment. Mean number of stage 4 RPD per eye was 9.33 +/− 5.41 before treatment (D1) and 10.0 +/− 5.15 after treatment.

**Figure 1:**
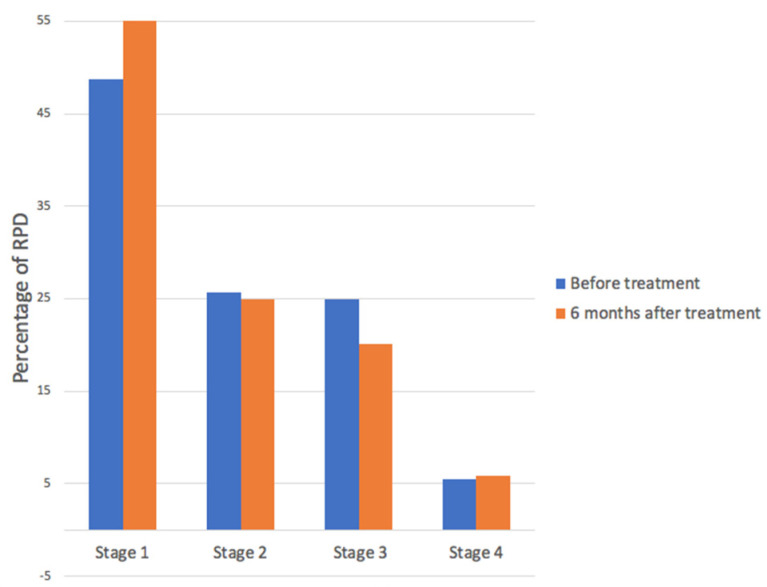
Percentage of reticular pseudodrusen (RPD) according to staging, before and 6 months after photobiomodulation treatment. Distribution of RPD among the RPD stages changed after the treatment. The number of stage 3 reticular pseudodrusen decreased while stage 1 reticular pseudodrusen increased after treatment.

**Figure 2:**
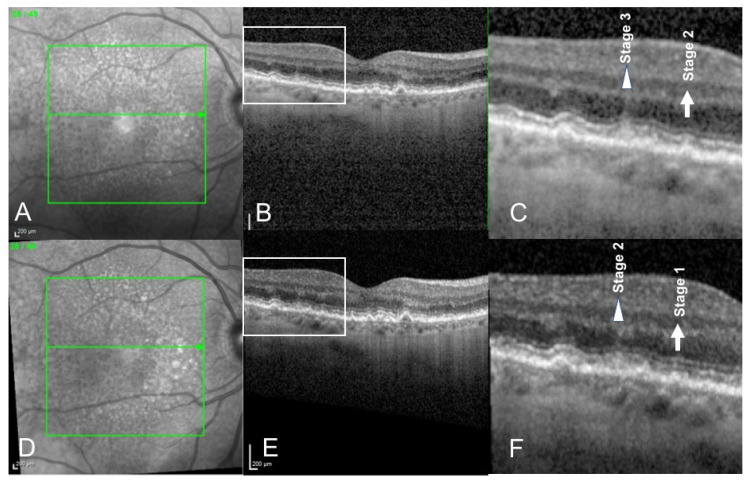
Spectral Domain Optical Coherence Tomography (SD-OCT) scans of a 76-year-old female patient presenting with reticular pseudodrusen, before (**A**–**C**) and 6 months after photobiomodulation treatment (**D**–**F**). Infrared imaging (**A**,**D**). SD-OCT scan showing redicular pseudodrusen before (**B**) and after (**E**) treatment. We can observe that a stage 3 reticular pseudodrusen (triangle, (**C**)) regressed into a stage 2 (triangle, (**F**)) reticular pseudodrusen 6 months after treatment. Similarly, a stage 2 reticular pseudodrusen (arrow (**C**)) regressed into a stage 1 reticular pseudodrusen (arrow, (**F**)) 6 months after photobiomodulation treatment.

**Figure 3:**
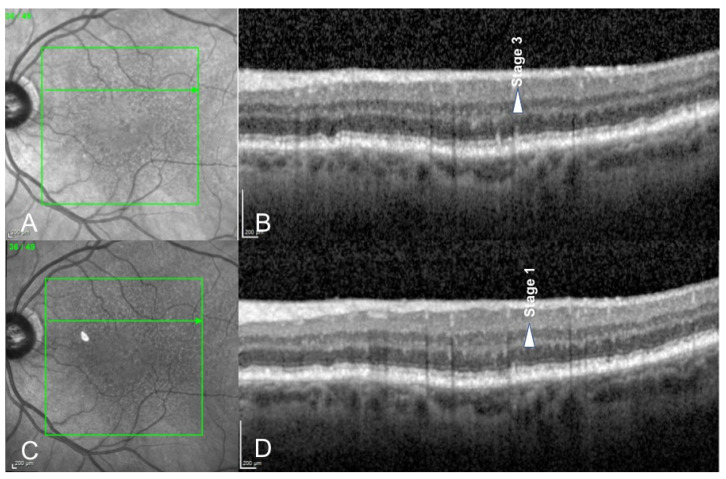
Spectral Domain Optical Coherence Tomography (SD-OCT) scans of a 76-year-old male patient before (**A**,**B**) and 6 months after photobiomodulation treatment (**C**,**D**). We can observe that a stage 3 reticular pseudodrusen (arrow, (**B**)) regressed into a stage 1 reticular pseudodrusen (arrow, (**D**)) after photobiomodulation treatment.

Changes in distribution according to pseudodrusen classification mostly concerned stages 1 and 3 RPD: Percentage of stage 1 RPD increased from 46% to 56% after treatment. Percentage of stage 3 RPD decreased from 20% to 13% after treatment. (Figure 1, Figure 2 and Figure 3).

Mean CMT measured 275.2 +/− 30.3 at baseline and 273.4 +/− 32.1 6 months after treatment. Mean choroidal subfoveal thickness measured 88.3 +/− 13.6 at baseline and 85.0 +/− 4.8 6 months after treatment.

Analyzing infrared and autofluorescence imaging, we did not find any development of atrophic lesions during the 6-month follow-up.

## 4. Discussion

We report the effect of photobiomodulation on drusen regression in patients with RPD. In our study, we observed that distribution of RPD among the RPD staging changed after the treatment (Figure 2 and Figure 3), although no statistical conclusions can be drawn due to the low number of patients. The number of stage 3 reticular pseudodrusen decreased while the number of stage 1 reticular pseudodrusen increased after treatment.

photobiomodulation had been found to be effective in patients suffering from dry AMD with functional and anatomical improvements [30]. photobiomodulation activates the mitochondrial respiratory chain components promoting cellular proliferation and cytoprotection. It counteracts inflammation, supports cell function and reduces oxidative damage [33]. Therefore, it has been used as a treatment in patients suffering from AMD in preclinical studies as well as in clinical studies.

Ivandic et al. studied low-level laser therapy in 203 patients (348 eyes) suffering from AMD, with or without cataracts. They used a semiconductor laser diode for transconjunctival irradiation of the macula. Visual acuity significantly increased in most of the eyes with (95%) and without cataracts (97%). Visual improvement lasted for 3 to 36 months after treatment with low-level laser therapy. Visual acuity remained unchanged in the control group. A decrease in the prevalence of scotomas, metamorphopsias and dyschromatopsias was noted. Patients suffering from neovascular AMD had less exsudation and less bleeding. No side effects were described in the low-level laser therapy treatment group [34].

Recently, studies led by LumiThera founders from the Toronto and Oak Ridge Study of Phobiomodulation (TORPA I and II) showed an increase in BCVA and contrast sensitivity after photobiomodulation treatment in patients suffering from dry AMD. TORPA I study [35] was a pilot prospective clinical study evaluating the effects of photobiomodulation on vision in patients with dry AMD. Primary outcomes measures were visual acuity, fixation stability and contrast sensitivity before and after treatment. A total of 18 eyes of nine patients were included in this pilot study. A statistically significant increase in visual acuity and contrast sensitivity was noted. However, no changes in fixation stability parameters were found. 

TORPA II [31] was an interventional longitudinal prospective study evaluating the effects of photobiomodulation in eyes with dry AMD. In total, 42 eyes were included in this study and treated with multiwavelength light emitting diode light with yellow, red and near-infrared wavelengths. Primary outcomes measures included changes in visual acuity, contrast sensitivity, drusen volume as well as central drusen thickness. Visual acuity significantly improved by 5.9 letters after 3 weeks of treatment and was maintained with a gain of 5.14 letters at 3 months. Significant improvement was also noted concerning contrast sensitivity after treatment. Drusen volume and central drusen thickness were also significantly reduced after photobiomodulation treatment, whereas overall central retinal thickness and retinal volume did not change. It was the first study showing improvements in anatomical outcomes in dry AMD patients following photobiomodulation therapy.

The LIGHTSITE I study [30] was a prospective double-masked clinical study evaluating the efficacy and safety of photobiomodulation treatment in patients with intermediate to dry AMD. It included 46 eyes of 30 patients with intermediate to advanced dry AMD who received a photobiomodulation treatment with the Valeda Light Delivery System (two series of treatment over a year, each series consisting of three treatment sessions per week for 3 to 4 weeks). Patients who received photobiomodulation treatment significantly improved their visual acuity by a mean of 4 letters at 1 month and at 7 months. Moreover, significant improvement was also noted in contrast sensitivity and quality of life. Central drusen thickness and central drusen volume were significantly reduced after treatment. No side effects nor adverse events were noted regarding the use of the device. This study supported the previous preclinical and clinical studies on photobiomodulation and suggested that photobiomodulation could clinically and anatomically be beneficial in patients suffering from dry AMD. However, the study included mostly patients with geographic atrophy, and no subgroup analysis was performed on patients with RPD without atrophy.

In their pilot study, Grewal et al. investigated the therapeutic effect of a 670 nm light exposure on visual functions and anatomical structures in healthy aging patients as well as in patients with AMD with and without RPD (subgroup of eight patients with RPD). They principally studied changes in rod-recovery time after a bright flash (primary outcome). They also described structural changes such as the thickness of the outer nuclear layer and the RPE-Bruch’s membrane complex. However, effects of photobiomodulation on RPD staging distribution have not been studied. They did not find any benefit of the 670 nm photobiomodulation on visual functions parameters in eyes with intermediate AMD with or without RPD [36]. 

As previously studied, RPD is dynamic and stages of RPD progress over time. In their study, Querques et al. analyzed RPD progression using SD-OCT over a mean period of 24 months. They found that 100% RPD graded as stage 1 progressed to stage 2, more than 80% of stage 2 RPD progressed to stage 3 and all stage RPD (100%) progressed to stage 4 [32]. In our case series, it seems that treated patients did not show this progression during the 6-month follow up. On the contrary, we observed a decrease in number of stage 3 RPD and an increase in stage 1 RPD. At the least, no progression of RPD into greater stages has been found, and anatomical progression of RPD was stabilized during the 6-month follow-up. This could be due to either the natural course of the disease and the short follow-up or to the effect of the photobiomodulation treatment that may possibly help stabilize progression of drusen. A control group would be necessary to better investigate the effect of the treatment.

Indeed, our study has several limitations. Among them is the low number of patients. The analysis was indeed purely descriptive since statistical analysis could not be performed due to the low number of patients. Moreover, this is a retrospective observational case series that did not include a control group nor was randomized. A further randomized double-arm study with a greater number of patients is needed to better evaluate the efficacy of the photobiomodulation treatment on drusen regression in patients presenting with RPD. The short follow-up is also a limitation. A longer follow-up is needed to better describe changes over time after photobiomodulation treatment. Anatomical changes seen on structural SD-OCT, but also functional changes such as visual acuity, would indeed be better described with a longer follow-up. Finally, drusen staging was inherently subjective and could form a bias. Finding a treatment to address the disease early in AMD patients could potentially slow the development of the disease and improve the visual prognosis. Therefore, other studies are needed to confirm or refute those results.

## 5. Conclusions

In conclusion, changes in RPD distribution were observed before and after treatment with photobiomodulation. The number of stage 3 reticular pseudodrusen decreased while the number of stage 1 reticular pseudodrusen increased after treatment. Further studies with a larger cohort and a longer follow-up are needed to better evaluate the effects of photobiomodulation treatment on reticular pseudodrusen.

## Data Availability

The data published in this research are available on request from the first author.
